# Prediabetes Subgroups, Type 2 Diabetes Risk, and Differential Effects of Preventive Interventions

**DOI:** 10.1210/clinem/dgaf350

**Published:** 2025-06-11

**Authors:** Jeanette M Stafford, Ramon Casanova, Byron C Jaeger, Yitbarek Demesie, Brian J Wells, Michael P Bancks

**Affiliations:** Wake Forest University School of Medicine, Winston-Salem, NC 27157, USA; Wake Forest University School of Medicine, Winston-Salem, NC 27157, USA; Wake Forest University School of Medicine, Winston-Salem, NC 27157, USA; Wake Forest University School of Medicine, Winston-Salem, NC 27157, USA; Wake Forest University School of Medicine, Winston-Salem, NC 27157, USA; Wake Forest University School of Medicine, Winston-Salem, NC 27157, USA

**Keywords:** prediabetes subgroups, type 2 diabetes prevention, intensive lifestyle intervention, metformin therapy

## Abstract

**Objective:**

Prior studies have subclassified type 2 diabetes using statistical clustering approaches with clinical data, but few have subclassified prediabetes and assessed the effects of preventive interventions. Our objective was to derive prediabetes subgroups based on clinical biomarkers and assess risk for incident diabetes and differential preventive intervention effects within the derived subgroups, with comparison to more simple modeling approaches.

**Methods:**

Baseline data for 3145 participants in the Diabetes Prevention Program trial were used to derive prediabetes subgroups using K-means clustering with data for 22 clinical biomarkers (sex-standardized). Cox proportional hazards regression was used to estimate hazard ratios (HRs) for diabetes and differential intervention effects (intensive lifestyle, metformin, or placebo) by prediabetes subgroups and to compare the clustering strategy to a model with clinical variables.

**Results:**

We identified 2 prediabetes subgroups characterized by severe insulin resistance with severe obesity (subgroup 1, 31% of sample) and moderate insulin resistance with overweight or obesity (subgroup 2, 69%). Subgroup 1 had a 58% higher risk for diabetes (HR: 1.58, 95% confidence interval: 1.31, 1.91) compared to subgroup 2. Randomization to lifestyle (compared to placebo) halved diabetes risk for both subgroups, while metformin provided greater benefit to subgroup 1 vs subgroup 2 (*P* for interaction <.05). A clinical variable model discriminated diabetes risk better than the clustering strategy.

**Conclusion:**

Pathophysiologically distinct prediabetes subgroups differ in risk for diabetes and preventive benefit from metformin. These results support distinct mechanisms of diabetes susceptibility; however, the use of clinical prediction models to guide treatment decisions may provide adequate risk profiling.

Type 2 diabetes is a disease of impaired glucose metabolism that results in high blood glucose levels (1). However, type 2 diabetes develops through multiple physiologic pathways, and these contribute to heterogeneity in metabolic etiology, clinical presentation, disease progression, organ system complications, and treatment response. Current methods for identifying individuals at high risk for or with new-onset type 2 diabetes rely solely on blood glucose measurements and do not capture the various physiological processes underlying the disease. Recent work to disentangle this heterogeneity has incorporated alternative physiological markers with data-driven methods to subclassify individuals with type 2 diabetes. Subgroups of type 2 diabetes have been identified that differ by genetic susceptibility; epigenetic, immune, and clinical profiles; risk for complications; and response to lifestyle, surgical, and pharmaceutical intervention (2-27).

Several studies have applied subgrouping to individuals without diabetes but with normal or impaired glucose tolerance (28-32). These demonstrate the utility of plasma metabolites (32), clinical characteristics (28-30), and polygenetic risk scores (31) to help substratify individuals into groups with differential metabolic profiles, risk for developing type 2 diabetes, and risk of complications. However, these prior studies were not conducted in the context of randomized interventions for diabetes prevention, and only 2 studies were restricted to individuals with prediabetic glucose levels. Assessment of prediabetes subgroups among randomized clinical trial populations enables the evaluation of interventions to help delay or prevent the onset of type 2 diabetes across and within subgroups and the potential to inform clinical guidance. To maximize the efficiency and cost-effectiveness of preventive interventions and allocation of healthcare resources, subgrouping individuals at risk for type 2 diabetes could prioritize those with prediabetes where interventions may yield a greater risk reduction. Further, the National Institute of Diabetes and Digestive and Kidney Diseases has called for a better understanding of heterogeneity in susceptibility to diabetes and response to therapies (33). It remains unknown whether prediabetes subgroups identified using a broad number of alternative physiological markers have differential benefit from lifestyle or pharmacological intervention. The objectives of this study were to identify prediabetes subgroups with unique physiological profiles and assess their risk for type 2 diabetes and differential prevention effects from intensive lifestyle intervention and metformin therapy.

## Research Design and Methods

This study was reviewed by the institutional review board of Wake Forest University School of Medicine and approved for exempt status (Exempt Protocol: IRB00091104). Prior institutional review board approval was obtained for protocols for the Diabetes Prevention Program (DPP) parent trial (clinicaltrials.gov ID: NCT00004992). Data for the DPP are publicly available and were accessed via https://repository.niddk.nih.gov/studies/dpp/.

### DPP

The DPP was a multicenter randomized clinical trial (34, 35). Briefly, from 1996 to 1999, 3819 individuals ages 25 to 85 years were enrolled at 27 clinical centers across the United States (1). Individuals who were at high risk for type 2 diabetes based on weight and glucose status were identified and consented to be screened and enrolled. Screening included an initial fasting glucose (FPG) level assessment and a subsequent 75-gram oral glucose tolerance test (OGTT). Eligibility criteria included age ≥25 years, body mass index (BMI) ≥24 kg/m^2^ (≥22 kg/m^2^ for Asian individuals), a FPG of 95 to a125 mg/dL (5.3-6.9 mmol/L, altered from 95-140 mg/dL in 1997 ), and a 2-hour OGTT glucose of 140 to 199 mg/dL (7.8-11.0 mmol/L). Individuals were excluded from DPP enrollment for any of the following: occurrence in the prior 6 months of myocardial infarction, symptoms of coronary heart disease, serious illness, or use of medications known to impair glucose tolerance (34). Race/ethnicity was self-reported based on the 1990 census questionnaire and collapsed into 4 categories for the public use data: White (58%), African American (20%), Hispanic (17%), and all other (5%).

DPP participants were randomized (stratified by clinical site) to 1 of 4 arms, described previously: intensive lifestyle intervention, standard care plus metformin, standard care plus troglitazone, or standard care plus placebo tablet (34, 35). The intensive lifestyle intervention was designed to achieve and maintain 7% weight loss through a healthy low-calorie and low-fat diet, maintaining moderate-intensity physical activity of ≥150 minutes weekly, and a 16-session behavioral change curriculum over 24 weeks designed to help set and achieve dietary and physical activity goals (monthly afterward). The metformin regimen was 850 mg twice daily with standard lifestyle recommendations and an annual 1-on-1 lifestyle session with a case manager. The troglitazone intervention was discontinued in 1998 due to concerns of liver toxicity, and postrandomization data from participants in this group were not included in the analysis (34). The placebo group received 1 tablet daily along with standard lifestyle recommendations and an annual 1-on-1 lifestyle session with a case manager.

### Clinical Clustering Variables

We started with 61 potential clinical, biological, or phenotypic variables to use for clustering (Supplementary Table S1) (36). From these variables, we excluded 39 from consideration for use in clustering. We excluded age at exam because it is not used in clinical diagnosis of diabetes and does not reflect age at diabetes onset as used in clustering of overt disease. We excluded sex-related hormones (n = 10) due to extreme differences in distribution by sex and lack of measurement in both sexes. We excluded 13 variables with >5% missing observations, setting this as a conservative threshold for missing observations to preserve data for multiple imputation of clustering variables. For the remaining 37 variables, we assessed between-variable correlations. We set a correlation threshold of 0.6, also used in other clustering studies (26). For variables with correlations exceeding 0.6 with 1 or more other variables (n = 23), we identified a single representative variable for use in clustering. This choice was based on maximizing the number of variables to keep and prioritizing variables that might be collected in a clinical setting and available in an electronic health record, which resulted in 15 variables being excluded. Data for the remaining 22 clinical variables or biomarkers were used in the clustering models. These variables included BMI, fasting proinsulin, fasting plasma glucose (PG), 30-minute PG, 2-hour PG, glycated hemoglobin (HbA1c), hemoglobin, homeostatic model of assessment (HOMA)-β cell function, platelet count, systolic blood pressure, low-density lipoprotein cholesterol, high-density lipoprotein cholesterol/triglyceride ratio, urine creatinine, urine albumin, serum alanine aminotransferase, fibrinogen, C-Reactive protein, tissue plasminogen activator, serum creatinine, serum bicarbonate, serum potassium, and serum sodium. Other studies aiming to subgroup prediabetes using phenotypic data have used fewer variables, typically limited to traditional predictors of type 2 diabetes risk (28-30). A broader set of variables was included for clustering in this study to help capture various physiological processes potentially underlying the development of diabetes.

### Primary Outcome: Diabetes Ascertainment

The primary outcome was incident diabetes, assumed to primarily be type 2 diabetes. In the DPP, diagnosis of type 2 diabetes was ascertained and defined by semiannual measurement of FPG ≥126 mg/dL (≥7.0 mmol/L) and annual post 75-gram OGTT 2-hour glucose ≥200 mg/dL (≥11.1 mmol/L) (34).

### Statistical Analysis

The analytic approach was 2-stage: (1) hard-clustering of participants at baseline and (2) assessing risk for developing diabetes. Participant exclusions and missing data are summarized in Supplementary Fig. S1 (36). We used publicly available deidentified data, and 3665 (66% women) of those enrolled in DPP agreed to have their data shared. Before clustering, individuals who met the current American Diabetes Association definition of diabetes via FPG or HbA1c (n = 513) at the time of DPP baseline and those with missing data for 3 or more clustering variables (n = 7) were excluded (1). The analytic sample for clustering was 3145. Fully conditional imputation was applied to handle missing values for 1 or 2 clustering variables for 144 individuals. Clustering variables were winsorized to the 1st and 99th percentile and then standardized within sex (mean = 0, SD = 1) to reduce the influence of extreme values and sex-related differences in the distribution on clustering. We applied k-means clustering to characterize subgroups of individuals with prediabetes at the time of randomization and assessed cluster numbers ranging from 2 to 6 groups. We calculated multiple metrics to assess cluster assignment, stability, separation, and similarity within cluster, including the Davies Bouldin and Silhouette Indices, which evaluate separation between clusters vs within cluster variability (37, 38). We also generated n = 1000 simple random samples at a 75% sampling rate and performed de novo clustering on each sample to calculate the Jaccard Index, a measure of similarity in an individual's cluster assignment across reclustered samples (39).

After establishing prediabetes subgroups at the time of randomization, we assessed the incidence of type 2 diabetes during the intervention phase. At this stage, we excluded individuals randomized to the troglitazone arm (n = 512). We used multivariable Cox proportional hazards regression during the model-building process, including sequential adjustment for prediabetes subgroup, randomization arm, an interaction between prediabetes subgroup and randomization arm, sex, age, HbA1c, HOMA-insulin resistance (IR), waist circumference, triglycerides, systolic blood pressure, race/ethnicity, and education.

We performed multiple sensitivity analyses to compare whether the use of clustering provided additional information on the risk for diabetes beyond simpler approaches. First, though randomization to troglitazone was balanced by prediabetes cluster, we used inverse probability of selection weighting to account for these exclusions in our analytic sample (n = 2633). Second, we identified absolute values for BMI (35.8 kg/m^2^) and HOMA-IR (7.9), respectively, that corresponded to the top 30% of the distribution (70th percentile) for each and created a binary categorization representing lower and higher risk for each clinical factor based on the cut-point. The top 30% was chosen to align with the proportion of the sample categorized to subgroup 1. This cut-point value categorization was compared to the prediabetes subgroup clusters with percent agreement to see if a simple categorization based on a single clinical risk factor yielded similar subgrouping. Third, we ran a Cox proportional hazards model for diabetes incidence when including adjustment for simple clinical variables age, sex, BMI, FPG, 2-hour glucose, randomization arm, and an interaction between randomization arm and each of the following: age, BMI, FG, and 2-hour glucose. These variables have previously been shown in the DPP to have differential intervention effects (40). This model was run with and without adjustment for the prediabetes subgroup, and results were compared across models to see if the use of prediabetes clustering improved discrimination of diabetes risk. SAS version 9.4 (SAS Institute, Cary, NC, USA) and R version 4.1.1 (The R Foundation) were used for analyses, and statistical tests were 2-sided with α = .05.

## Results

Of those included in the clustering analysis, the mean age was 50.5 years, 67% were women, 62% were White, 16% were Black, and 17% were Hispanic (Supplementary Table S2) (36). Those excluded were more likely to be Black, older, current smokers, and have higher blood glucose levels, BMI, blood pressure, and HOMA-IR, compared to those included. Most, but not all, cluster model statistics supported n = 2 as the optimal number of clustering subgroups, and after combined consideration of these metrics, we proceeded with 2 prediabetes subgroups for the primary analysis (Supplementary Table S3) (36). The Jaccard Index for n = 2 subgroups was 0.83 overall, 0.52 for subgroup 1 (n = 975), and 0.97 for subgroup 2 (n = 2170). We did not assess for statistical differences in cluster characteristics between subgroups, and all comparisons of baseline (randomization) characteristics are qualitative. At randomization, the prediabetes subgroups differed in multiple metabolic clinical factors (Table 1 and Supplementary Table S4) (36). Subgroup 1 (severe insulin resistance with severe obesity), 31% of the sample, had higher BMI, fasting glucose, and measures of HOMA-IR and HOMA-β cell function and lower values for the ratio of high-density lipoprotein cholesterol to triglycerides compared to subgroup 2 (moderate insulin resistance with overweight or obesity). Additional clustering factors with different distributions between subgroups included proinsulin, fibrinogen, C-reactive protein, and tissue plasminogen activator, while 30- and 120-minute glucose, HbA1c, markers of blood cell function, blood electrolyte balance and acidity, and liver and kidney function did not appear to differ substantively. The proportion of individuals with prediabetes levels of HbA1c and BMI ≥30 kg/m^2^ was higher in subgroup 1 than subgroup 2, respectively. Subgroup 1 had a lower mean age at randomization, while the subgroups did not appear to differ by sociodemographic factors such as sex, race/ethnicity, and educational attainment. Smoking status was similar between subgroups, while subgroup 1 had a greater percentage of persons who did not regularly consume alcohol. Randomization arm allocation was also not substantively different by prediabetes subgroup.

**Table 1.** Baseline characteristics of Diabetes Prevention Program participants (n = 3145) according to k-means clustering prediabetes subgroup

Characteristic*^a^*	Prediabetes subgroup 1*^b^*	Prediabetes subgroup 2*^b^*
Number, n (% total)	975 (31)	2170 (69)
Age, years (SD)	48.24 (9.76)	51.57 (10.58)
Age range, years	25.7, 79.0	25.4, 84.4
Female sex, n (%)	677 (69.4%)	1428 (65.8%)
Race/ethnicity, n (%)		
Non-Hispanic White	601 (61.6)	1346 (62.0)
Non-Hispanic Black	167 (17.1)	331 (15.3)
Hispanic	177 (18.2)	359 (16.5)
Other	30 (3.1)	134 (6.2)
Educational attainment, n (%)		
<High school	63 (6.5)	149 (6.9)
High school graduate	195 (20.0)	354 (16.3)
Some college	465 (47.7)	1055 (48.6)
≥College graduate	252 (25.8)	612 (28.2)
Fasting glucose, mg/dL (SD)	108.9 (7.38)	105.1 (6.07)
Fasting glucose, mmol/L (SD)	6.1 (0.4)	5.8 (0.3)
Glycated hemoglobin, % (SD)	5.87 (0.37)	5.73 (0.41)
Glycated hemoglobin, mmol/mol (SD)	41 (4.0)	39 (4.5)
Prediabetes glycated hemoglobin (≥39 mmol/mol), n (%)	736 (75.5)	1339 (61.7)
HOMA-insulin resistance, median (IQR)	9.3 (7.2, 12.3)	5.1 (3.5, 6.7)
HOMA-β cell function, median (IQR)	277 (210, 377)	167 (118, 224)
Body mass index, kg/m^2^ (SD)	39.2 (6.9)	31.1 (4.4)
Body mass index (category), n (%)		
<25 kg/m^2^	2 (0.2)	90 (4.2)
25-29.9 kg/m^2^	57 (5.8)	908 (41.8)
≥30 kg/m^2^	916 (94.0)	1172 (54.0)
Low-density lipoprotein cholesterol, mg/dL (SD)	121.5 (32.2)	125.5 (31.7)
High-density lipoprotein cholesterol, mg/dL (SD)	41.8 (10.3)	47.3 (12.1)
Triglycerides, mg/dL, median (IQR)	162 (114, 225)	136 (96, 197)
Systolic blood pressure, mmHg (SD)	127 (14)	122 (14)
Diastolic blood pressure, mmHg (SD)	80 (10)	77 (9)
Smoking status, n (%)		
Never	576 (59.1)	1287 (59.3)
Former	325 (33.3)	766 (35.3)
Current	74 (7.6)	117 (5.4)
Alcohol consumption, n (%)		
No regular	591 (60.6)	1101 (50.7)
≤1 drink daily	356 (36.5)	985 (45.4)
>1 drink daily	28 (2.9)	84 (3.9)
DPP randomization arm, n (%)		
Placebo	269 (27.6)	610 (28.1)
Metformin	265 (27.2)	603 (27.8)
Lifestyle	293 (30.1)	593 (27.3)
Troglitazone	148 (15.2)	364 (16.8)

Abbreviations: HOMA, homeostatic model of assessment; IQR, interquartile range.

^*a*^Values are means (SD), median (IQR), or number (%)

^*b*^Prediabetes subgroup 1 is characterized by severe insulin resistance with severe obesity, and prediabetes subgroup 2 is characterized by moderate insulin resistance with overweight or obesity.

### Diabetes Incidence

**Figure 1.**
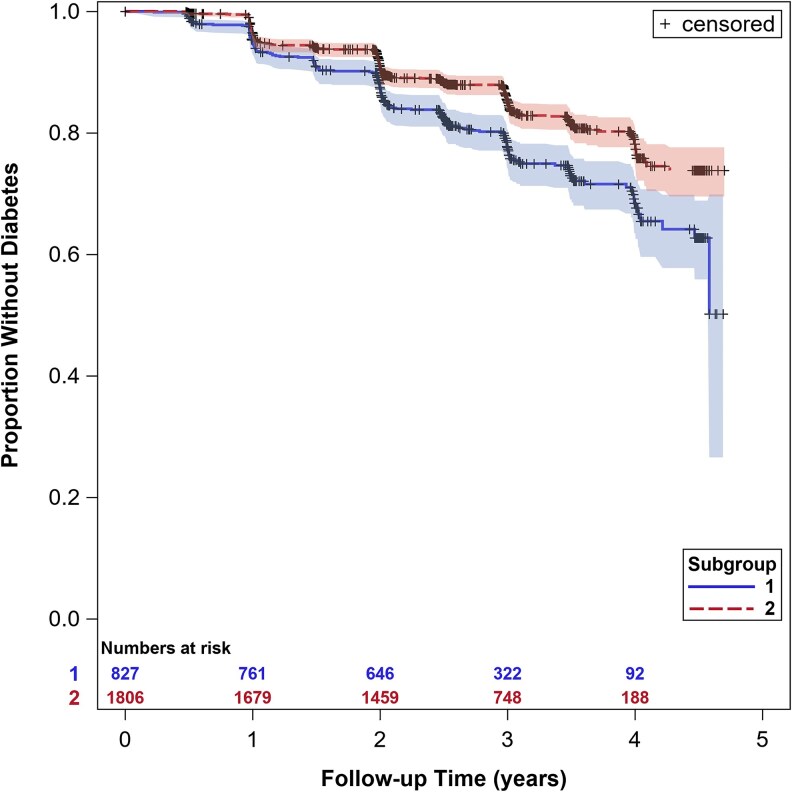
Kaplan–Meier survival plot for type 2 diabetes according to baseline prediabetes subgroup in the Diabetes Prevention Program.

There were 462 incident cases of diabetes (17.5% cumulative incidence) among the 2633 participants (7283.6 person-years) included in the prospective analysis. The diabetes incidence rate per 100 years was 8.4 for subgroup 1, with 22.9% cumulative incidence and median follow-up time 2.63 years, while subgroup 2 had an incidence rate of 5.4 incident cases per 100 years with 15.1% cumulative incidence and median follow-up time 2.97 years (Fig. 1). Before assessing for differential intervention effects, the hazard ratio (HR) for incident diabetes was 1.58 [95% confidence interval (CI): 1.31, 1.91] for prediabetes subgroup 1 compared to subgroup 2 after adjustment for randomization arm, sex, and age (Table 2). When including inverse probability of selection weighting to account for the exclusion of participants randomized to the troglitazone arm, this HR was 1.66 (95% CI: 1.38, 1.99).

**Table 2.** Incidence of type 2 diabetes and HRs (95% confidence intervals) according to prediabetes subgroup in the Diabetes Prevention Program (n = 2633)

	Prediabetes subgroup 1	Prediabetes subgroup 2
Number at risk, n	827	1806
Incident diabetes events, n	189	273
Total person time, years	2252.1	5031.5
Median person time, years (range: 25%, 75%)	2.6 (2.0, 3.5)	3.0 (2.3, 3.5)
Diabetes HR (95%) model 1 without interaction and without IPSW	1.58 (1.31, 1.91)	Reference
Diabetes HR (95%) model 1 without interaction and with IPSW	1.66 (1.38, 1.99)	Reference
Model 1*^a^* without IPSW		
*P* for interaction (intervention*^a^* subgroup)	.0233	
Intention to treat HR for diabetes (95%)	HR (95%)	HR (95%)
Lifestyle vs placebo (reference)	0.49 (0.35, 0.69)	0.46 (0.33, 0.63)
Metformin vs placebo (reference)	0.50 (0.35, 0.71)	0.86 (0.66, 1.12)
Lifestyle vs metformin (reference)	0.98 (0.67, 1.45)	0.53 (0.38, 0.74)
Concordance statistic	0.6106	
		
Model 2*^b^* without IPSW		
*P* for interaction (intervention*^a^* subgroup)	.0117	
Intention to treat HR for diabetes (95%)	HR (95%)	HR (95%)
Lifestyle vs placebo (reference)	0.47 (0.33, 0.66)	0.46 (0.33, 0.63)
Metformin vs placebo (reference)	0.47 (0.33, 0.67)	0.87 (0.67, 1.14)
Lifestyle vs metformin (reference)	0.99 (0.67, 1.47)	0.52 (0.38, 0.72)
Concordance statistic	0.6195	
		
Model 3*^c^* without IPSW		
*P* for interaction (intervention*^a^* subgroup)	.0124	
Intention to treat HR for diabetes (95%)	HR (95%)	HR (95%)
Lifestyle vs placebo (reference)	0.46 (0.33, 0.65)	0.45 (0.33, 0.62)
Metformin vs placebo (reference)	0.47 (0.33, 0.67)	0.87 (0.67, 1.14)
Lifestyle vs metformin (reference)	0.98 (0.66, 1.45)	0.52 (0.37, 0.72)
Concordance statistic	0.6216	

Abbreviations: HR, hazard ratio; IPSW, inverse probability of selection weighting.

^*a*^Model 1 includes adjustment for prediabetes subgroup, randomization arm, sex, age, and an interaction term (prediabetes subgroup * randomization arm).

^*b*^Model 2 includes characteristics in model 1 plus glycated hemoglobin, homeostatic model of assessment-insulin resistance, waist circumference, triglycerides, and systolic blood pressure.

^*c*^Model 3 includes characteristics in model 2 plus race/ethnicity and education.

### Differential Intervention Effects

**Figure 2.**
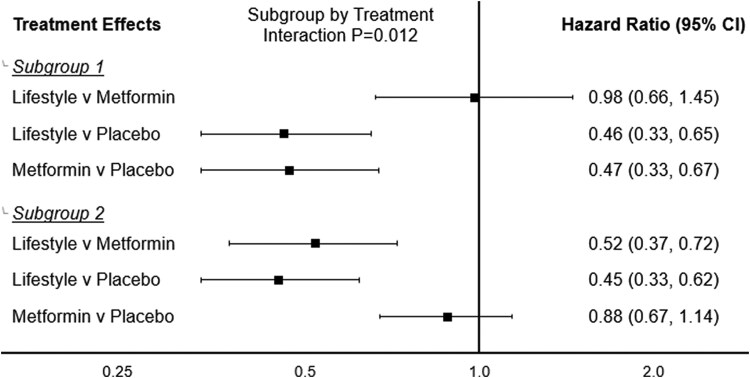
Hazard ratio for incident type 2 diabetes according to randomization arm within baseline prediabetes subgroup after adjustment for sex, age, glycated hemoglobin, homeostatic model of assessment-insulin resistance, waist circumference, triglycerides, systolic blood pressure, race/ethnicity, and education without inverse-probability of selection weighting (model 3 from Table 2).

We detected heterogeneity in the age- and sex-adjusted effect of interventions across prediabetes subgroups (subgroup by intervention *P* for interaction = .0233). Going forward, we continued with this interaction term in models that estimated intervention effects by prediabetes subgroup. Within both prediabetes subgroups, randomization to lifestyle was associated with a >50% reduction in risk for diabetes compared to placebo (Table 2). Among prediabetes subgroup 1, randomization to metformin was associated with a 50% reduction in diabetes risk (HR: 0.50; 95% CI: 0.35, 0.71), while in prediabetes subgroup 2, the HR was 0.86 (95% CI: 0.66, 1.12). When comparing randomization to lifestyle vs metformin, the diabetes HR was 0.98 (95% CI: 0.67, 1.45) among subgroup 1 and 0.53 (95% CI: 0.38, 0.74) among subgroup 2. These estimates did not change considerably after further adjustment for HbA1c, HOMA-IR, waist circumference, triglycerides, systolic blood pressure, race/ethnicity, and educational attainment (Table 2 and Fig. 2). The concordance statistic from our final adjusted model from our primary analysis that assessed prediabetes subgroups as the main effect, including interaction with the randomization arm and adjustment for clinical and demographic variables, was 0.6216 (subgroup by intervention *P* for interaction = .0124).

### Sensitivity Analyses

We observed kappa coefficients of 0.51 and 0.54 when applying binary categorization based on cut-point values at the 70th percentile for BMI and HOMA-IR (high/low), respectively, compared to prediabetes subgroup membership (Supplementary Table S5) (36). Observed vs expected percent agreement was 20% points higher for BMI and HOMA-IR binary categorization comparisons to the prediabetes subgroup. These metrics were lower when comparing the BMI with HOMA-IR binary categories.

The concordance statistic from a Cox model for diabetes incidence that included adjustment for only age, sex, BMI, FPG, 2-hour glucose, randomization arm, and interactions between randomization arm and age, BMI, FG, and 2-hour glucose each was 0.7255. When the prediabetes subgroup was added to this simple clinical model with interactions, the concordance statistic was 0.7260, and the HR comparing prediabetes subgroup 1 to subgroup 2 was 0.92 (95% CI: 0.72, 1.17). We did not detect heterogeneous intervention effects across prediabetes subgroups in this model (*P* = .26).

## Discussion

In this secondary data analysis of the DPP, we used k-means clustering approaches coupled with data for 22 clinical variables to derive prediabetes subgroups. Among this population with impaired glucose tolerance and at high risk for diabetes, we identified 2 prediabetes subgroups at the time of randomization that were characterized by severe insulin resistance with severe obesity and moderate insulin resistance with overweight or obesity. The effect of the lifestyle and metformin interventions differed by prediabetes subgroup before accounting for the known individual clinical risk factor differential intervention effects in this sample. For both prediabetes subgroups, intensive lifestyle modification drastically reduced the risk of diabetes compared to a strategy of standard lifestyle recommendations (placebo). Among individuals in the subgroup with severe insulin resistance with severe obesity, in about one-third of participants, metformin therapy was similarly effective at halving the risk for diabetes compared to placebo, with almost no difference in effect compared to intensive lifestyle modification. Metformin therapy was less effective overall among the prediabetes subgroup with moderate insulin resistance with overweight or obesity. In contrast, lifestyle modification was considerably more effective at reducing risk for diabetes compared to metformin among this subgroup, which was almost two-thirds of the study sample.

For the subgroup where there was no discernable difference in diabetes prevention for intensive lifestyle modification compared to metformin, it is likely advisable that individuals should maintain lifestyle modification of diet and physical activity for weight loss (1), possibly at a moderate rather than intensive rigor and duration, as metformin therapy in combination with intensive lifestyle intervention was not assessed in DPP. While not established, it is unlikely that metformin with intensive lifestyle modification would cause harm.

An important aspect of our work was to compare the utility of prediabetes clustering of manifold clinical variables with simpler approaches. After clustering, we observed that the 2 prediabetes subgroups were qualitatively distinguished by measures of insulin resistance and overweight/obesity. To this point, qualitative differences between prediabetes subgroups were observed for less than a third of the 22 clinical variables used for clustering, suggesting they were not informative for subgrouping. When we compared adjusted risk models with the prediabetes subgroup as the main effect and with interactions with the DPP interventions to a model with simple clinical variables, the clinical variable model performed well at discriminating diabetes risk, and the addition of prediabetes subgroups did not discernably improve performance. Further, the clinical variable model allows for specifying interactions between clinical variables and the DPP interventions to precisely target a prevention strategy to a unique clinical profile. We also compared overlap in prediabetes subgroup membership with binary categories of lower and higher risk individuals based on absolute values for clinical factors of insulin resistance and BMI. Results from these supplementary analyses suggest that categorizing disease susceptibility and benefit from guideline-recommended preventive interventions based on a single clinical factor can produce moderate agreement with more complex data-driven approaches. To this end, the original trial prespecified subgroup analyses by cut-points for BMI, fasting, and 2-hour glucose, which our results do align with (ie, stronger effect of metformin with higher BMI), and are used in clinical settings (40). Also, the use of discrete cut-points for measures of insulin secretion and resistance and liver fat may help to partition individuals into higher and lower risk groups and target a more intensive lifestyle modification program to high-risk individuals for additional benefit (41). Characterizing risk based on isolated or concomitant impaired fasting glucose, impaired glucose tolerance, and prediabetes HbA1c levels may help identify individuals who may achieve even greater risk reduction from intensive lifestyle interventions (42).

Our work helps broaden the understanding from prior clustering of individuals with prediabetes. Two groups have used approaches to subgroup prediabetes based on underlying genetic risk or metabolomic profiles (31, 32). Metabolomics and genetics data were not available in the DPP public use data and could not inform our clustering strategy. Three studies have used clinical data to partition subgroups of individuals with normal glucose tolerance or prediabetes glucose levels and assess risk for diabetes, and all 3 groups identified 6 prediabetes clusters, none of which exceeded 26% of the study sample (28-30). The study populations were uniquely European, Mexican, and Chinese descent and varied in the number of clinical variables used (8 to 12), including from the following: sex, family history of diabetes, educational attainment, and measures of glucose metabolism and response; blood pressure; body mass and anthropometry; insulin resistance and β-cell function, lipids; liver enzymes; and imaging-based measures of body fat distribution and liver fat content (28). All 3 studies observed that the highest risk for diabetes was among subgroups with the worst profiles for β-cell failure, insulin resistance, and fatty liver or liver dysfunction. Compared to these 3 studies, the DPP trial population includes a narrower and high risk for diabetes definition of individuals with prediabetes: those with both impaired fasting glucose and impaired glucose tolerance. Further, a high percentage of participants in both subgroups in our study met the criteria for prediabetes via HbA1c. This enrichment of a high risk for diabetes population may explain why we observed that 2 subgroups best fit this study sample, rather than 6. Additionally, none of these prior studies applied prediabetes subgrouping in the context of randomized preventive interventions. As a result, they could not assess differential prevention benefits by subgroup, which could help disentangle the heterogeneity underlying type 2 diabetes pathophysiology and support the development of pathophysiology-based precision medicine approaches to prevention. Stroebel et al applied clustering among a subset of DPP participants using clinical data similar to what we used in addition to imaging measures from computed tomography (43). The silhouette statistics supported 2 subgroups that best fit the data, similar to our findings in DPP, though investigators chose 5 subgroups for longitudinal assessment due to greater separation in cluster profiles.

Several limitations of our work should be considered. First, we assessed prediabetes subgroups, and differential intervention effects among these subgroups, that were not considered during the DPP trial design. While randomization was balanced by a prediabetes subgroup, this work is exploratory, and our analyses are post hoc and require confirmation in other studies. Second, we included numerous variables in our clustering model to capture various physiological processes that could not be otherwise be characterized with fewer variables, but these variables are not regularly collected in clinical settings. Also, we were unable to include specific measures of fat distribution or tissue fat content because they were not measured on the entire randomized sample. The data and clustering methods used here may not be optimal to disentangle heterogeneity in diabetes susceptibility. For example, it is possible that the results were influenced by the dimensionality of the dataset (22 clustering variables). The generalization of results in the current analysis to external populations should be investigated further, as clustering techniques may produce results that do not generalize beyond their internal data.

In conclusion, we applied data-driven approaches to partition individuals at high risk for diabetes into prediabetes subgroups among a large racially/ethnically diverse US adult population enrolled in a randomized trial of multiple diabetes preventive interventions. These prediabetes subgroups were characterized by differences in clinical measures of obesity and insulin resistance at baseline, which corresponded to differential risk for developing diabetes as well as differential response to interventions to prevent diabetes. For most individuals at high risk for diabetes, intensive lifestyle intervention is successful at diabetes prevention. For a small subset with severe insulin resistance and obesity and very high risk for diabetes, metformin therapy is also beneficial for diabetes prevention. Despite these advancements in prediabetes subgrouping, our results did not support using similar prediabetes clustering approaches in place of simpler methods and specific clinical measures to characterize risk profiles, estimate diabetes risk, and target preventive interventions.

## Data Availability

The Diabetes Prevention Program (DPP) was conducted by the DPP Investigators and supported by the National Institute of Diabetes and Digestive and Kidney Diseases (NIDDK). The data from the DPP (doi:10.58020/3hw5-cf91) reported here were supplied by the NIDDK Central Repository (NIDDK-CR) and are available for request at https://repository.niddk.nih.gov/studies/dpp/. This manuscript was not prepared under the auspices of the DPP study and does not necessarily reflect the opinions or views of the DPP study, NIDDK-CR, or NIDDK.

## Prior Presentation

M.B. presented preliminary findings of this project at the American Diabetes Association Scientific Sessions as a poster presentation, June 21-24, 2024.
